# Resveratrol ameliorates cardiac dysfunction induced by pressure overload in rats via structural protection and modulation of Ca^2+^ cycling proteins

**DOI:** 10.1186/s12967-014-0323-x

**Published:** 2014-11-26

**Authors:** Qi Dong, Zhiye Wu, Xiaoyun Li, Jianyun Yan, Luning Zhao, Chuntao Yang, Junjiang Lu, Ju Deng, Minsheng Chen

**Affiliations:** Department of Physiology, Guangzhou Medical University, Guangzhou, 510182 China; Guangzhou Institute of Cardiovascular Disease, Guangzhou, 510260 China; Southern Medical University, 1838 Guang zhou da dao bei, Guangzhou, 510515 China; Department of Cardiology, The Second Hospital Affiliated with Guangzhou Medical University, Guangzhou, 510260 China; Experimental Medical Research Center, Guangzhou Medical University, Guangzhou, 510182 China; Department of Neurology, The Fifth Hospital Affiliated with Zunyi Medical College, Zhuhai, 519100 China

**Keywords:** Resveratrol, Heart failure, Hypertrophy, Ultrastructure, Ca^2+^ handling proteins

## Abstract

**Background:**

Cardiac hypertrophy is a compensatory stage of the heart in response to stress such as pressure overload (PO), which can develop into heart failure (HF) if left untreated. Resveratrol has been reported to prevent the development of hypertrophy and contractile dysfunction induced by PO. However, other studies found that resveratrol treatment for a longer period of time failed to regress cardiac hypertrophy. The aim of this study is to determine the timing of resveratrol treatment to achieve antihypertrophic effect and investigate whether resveratrol prevents the development of HF through preservation of myocardium structure and modulation of Ca^2+^ handling proteins.

**Methods:**

To generate rats with cardiac hypertrophy, male Sprague–Dawley rats were subjected to PO (aortic banding procedure) for 4 weeks. Sham-operated animals served as controls. Rats with cardiac hypertrophy were given resveratrol (4 mg/kg/day) for 4, 6, and 8 weeks, respectively. Histological and echocardiographic analysis and transmission electron microscopy were performed to assess cardiac structure and function. The levels of Ca^2+^ handling proteins were measured by western blot analysis.

**Results:**

Histological analysis showed that resveratrol treatment regressed developed cardiac hypertrophy at 8 and 10 weeks postsurgery, but not at 12 weeks. However, resveratrol strongly and continuously prevented the development of cardiac dysfunction and dilation of cardiac chamber as evaluated by echocardiography and H&E staining of heart cross-sections. In addition, PO-induced cardiac fibrosis was completely inhibited by resveratrol treatment. Resveratrol markedly prevented the disrupted myocardium but partially rescued mitochondrial abnormality in banded rats. Moreover, resveratrol prevented the alteration of Ca^2+^ handling proteins induced by aortic banding, including downregulation of sarcoplasmic reticulum Ca^2+^ ATPase _2_ (SERCA_2_) and ryanodine receptor _2_ (RyR_2_), hypophosphorylated phospholamban (PLB), upregulation of Na^+^/Ca^2+^-exchangers (NCX_1_) and increased expression and phosphorylation of Ca^2+^/calmodulin -dependent protein kinase II (CaMKII). Moreover, resveratrol alleviated the decreased SERCA activity induced by aortic banding.

**Conclusions:**

Resveratrol effectively prevented the transition from compensatory to decompensatory stage of cardiac hypertrophy induced by PO, but this effect is dependent on the timing of treatment. We suggest that resveratrol may exert beneficial effects on cardiac hypertrophy through protection of cardiac structure and modulation of Ca^2+^ handling proteins.

**Electronic supplementary material:**

The online version of this article (doi:10.1186/s12967-014-0323-x) contains supplementary material, which is available to authorized users.

## Background

Heart failure (HF) is a major cause of hospitalization and mortality worldwide [1]. Increased hemodynamic load is one of the major contributors to the development of HF. Cardiac hypertrophy is considered to be an adaptive response to pressure or volume overload, which is characterized by the enlargement of heart muscle. Although the initial phase is beneficial in maintaining cardiac function, prolonged hypertrophy will lead to deleterious consequences and eventually HF. Nowadays, many medicines, such as β-adrenergic receptor blockers, angiotensin-converting enzyme inhibitors, angiotensin-receptor blockers and diuretics, have been universally used to treat cardiac hypertrophy and HF. These medicines can be effective to partially relieve the symptoms of HF, but cannot reverse the progression of HF. In fact, mortality rates of HF still approach about 20% per year [1]. Therefore, it is critically important to explore alternative approaches to prevent or reverse the progression of cardiac hypertrophy and HF.

Previous studies including The Dietary Approaches to Stop Hypertension (DASH) trial and Lyon Diet Heart Study have demonstrated that increased intake of fruits and vegetables lowered blood pressure in patients with hypertension, reduced the incidence of cardiovascular diseases, and improved survival after myocardial infarction [2]. Furthermore, several studies reported that resveratrol (trans-3, 5, 49- trihydroxystilbene), which is a phenolic phytoalexin and presents in grapes, berries and red wine, alleviated contractile dysfunction, reversed pressure overload (PO)-induced cardiac hypertrophy [1,3,4] and reduced cardiovascular mortality [5]. Wojciechowski P [1] and Juric D [2] found that resveratrol treatment for 2 weeks regressed cardiac hypertrophy in aortic banded rats. However, Ste’phanie Rimbaud demonstrated that resveratrol treatment for 8 weeks did not counteract cardiac hypertrophy in the Dahl salt-sensitive (DSS) rats fed with a high-salt diet (HS-NT) [5]. These inconsistent results indicated that resveratrol may reverse cardiac hypertrophy in a timing-dependant manner. In this study, we investigated the optimal timing of resveratrol treatment to achieve antihypertrophic effect in rats subjected to PO.

Additionally, it is well demonstrated that resveratrol protects against the development of HF by improving contractile functions [1-5]. However, precise mechanisms of its action remain unclear. The normal myocardial structure is essential for cardiac contraction. The most prominent ultrastructural alteration in HF is the loss of contractile elements, which significantly results in reduced cardiac function. Therefore, it is worthwhile to investigate whether resveratrol ameliorates ultrastructural abnormalities in failing hearts induced by PO.

Ca^2+^ cycling, a critical process referring to the mobilization of intracellular Ca^2+^ in excitation-contraction (EC) coupling, determines cardiac contractility and relaxation. It is now generally accepted that defective Ca^2+^ handling proteins in the cycling play an important role in HF pathophysiology. The key defects in Ca^2+^ cycling occur at the level of the sarcoplasmic reticulum (SR), a Ca^2+^ storage bulk in muscle. The SR Ca^2+^ release channel (ryanodine receptor _2_, RyR_2_) is oxidized, nitrosylated, and hyperphosphorylated, resulting in “leaky” channels and depletion of Ca^2+^ from the SR in failing hearts. Downregulated SR Ca^2+^ ATPase _2a_ (SERCA_2a_) and hypophosphorylated phospholamban (PLB) contribute to impaired SR Ca^2+^ uptake that conspires with leaky RyR_2_ to deplete SR Ca^2+^ in failing hearts [6]. Accordingly, the other goal of this study is to verify the hypothesis that resveratrol fixes Ca^2+^ handling proteins in SR to preserve contractile function in rats subjected to PO.

## Methods

### Animal model

The investigation conforms to the Guide for the Care and Use of Laboratory Animals published by the US National Institutes of Health (NIH Publication No. 85–23, revised 1985). Male Sprague–Dawley rats weighing 80-100 g were obtained from Central Animal Care Services at Southern Medical University and subjected to the abdominal aortic banding procedure for induction of PO. Briefly, rats were kept in a room with constant temperature of 26°C, humidity of 55%, and a 12 h light: 12 h dark cycle throughout the study. Animals were given standard rat chow and tap water ad libitum. All rats were anesthetized for surgery with 10% chloral hydrate (3 ml/kg). A midline laparotomy was performed, and the suprarenal abdominal aorta was exposed. The aorta between the branches of celiac artery and anterior mesenteric artery was tied off by a 4–0 silk suture with a blunt 24-gauge needle as a guide. Sham-operated rats were served as controls and subjected to the same surgeries except for the creation of the aortic band. Resveratrol treatment started from 4 weeks postsurgery when LV hypertrophy ocurred, as indicated by the results of echocardiography, and aortic banded rats were divided into treated and untreated subgroups. Resveratrol (Sigma, 4 mg/kg/d) was suspended in 0.5% carboxymethylcellulose (CMC, Sigma) dissolved in 0.9% saline. This solution was administered to treated rats by oral gavages for 4, 6 and 8 weeks, respectively. 0.9% saline was used as a vehicle to treat sham and banded animals.

### Echocardiography

At 4, 8, 10 and 12 weeks postsurgery, rats from all groups were weighed and anesthetized with chloral hydrate. Transthoracic two-dimensionally guided M-mode echocardiography was performed using an IE33 echocardiographic system (Philips Medical Systems, Nederland B.V) equipped with a 15-MHz (s12) transducer. The following parameters were measured: percentage of left ventricular (LV) fractional shortening (FS), LV ejection fraction (EF), maximal velocity through left ventricular outflow tract (Vmax), Cardiac output, LV pressure half-time, LV internal dimensions at both diastole and systole (LVIDd and LVIDs, respectively), LV posterior wall dimensions at both diastole and systole (LVPWd and LVPWs, respectively), and interventricular septal dimensions at both diastole and systole (IVSd and IVSs, respectively).

### Organ weight and histological analysis

Body weight, heart and LV with septum were weighed, and the LV mass-to-body weight ratio (LVm/BW) was calculated. LV samples were fixed in 10% neutral buffered formalin, embedded with liquid paraffin, and sectioned into 6 μm in thickness. Myocyte cross-sectional area was used for the evaluation of the degree of LV hypertrophy. Briefly, sections were stained with hematoxylin and eosin (HE) and examined under a light microscope (Nikon ECLIPSE Ti-U). Five random fields from each of 4 sections per animal were analyzed, and 10 ~ 15 myocytes per section were measured. The quantification of diameter and area of myocytes were determined with Image Pro Plus 6.0 (Media Cybernetics, Carlsbad, CA). To assess fibrosis, sections were stained with a Masson trichrome kit (Baso, BA4079) according to manufacturer’s instructions and stained sections were examined under a light microscope. Interstitial and perivascular fibrosis content were quantified in tissue sections using Image Pro Plus 6.0 as the percentage of connective tissue per cross-sectional surface area. The cross sectional area of LV chamber was also measured.

### Transmission Electron Microscopy (TEM)

Hearts were perfused and fixed in 2.5% glutaraldehyde in 0.1 M sodium cacodylate buffer followed by post-fixation with 2% osmium tetroxide in 0.1 M phosphate buffer (pH 7.4) for 2 h at room temperature. The tissue cubes were then dehydrated in an ascending series of ethanol solutions, followed by acetone dehydration. The tissue cubes were embedded with Embed 812 resin overnight at 37°C. 40 ~ 60 nm ultrathin sections were cut on an ultramicrotome, picked up on grids, and stained with a solution of lead citrate and uranyl acetate. The sections were then observed, and photographs were taken under a Hitachi 7650 electron microscope.

### Western blot analysis

LV tissues frozen in liquid nitrogen were pulverized and homogenized in a lysis buffer containing 20 mM Tris–HCl (pH 7.5), 150 mM NaCl, 1 mM Na_2_EDTA, 1 mM EGTA, 1% Triton, 2.5 mM sodium pyrophosphate, 1 mM beta-glycerophosphate, 1 mM Na_3_VO_4_, 1 μg/ml leupeptin (20 ml/g tissue) and 1 mM phenylmethylsulfonyl fluoride. Protein samples (20 ~ 25 μg) were separated by sodium dodecyl sulfate polyacrylamide gel electrophoresis (SDS-PAGE) and transferred to polyvinylidene difluoride (PVDF) membranes. PVDF membranes were probed with the following primary antibodies: anti-Ca^2+^/calmodulin-dependent protein kinase II (CaMKII) antibody (Abcam, 1:1000 dilution), anti-phospho-CaMKII (Thr286) antibody (p-CaMKII, CST, 1:1000 dilution), anti-PLB antibody (Abcam, 1:1000 dilution), anti-phospho-PLB (S16) antibody (p-PLB, Abcam, 1:1500 dilution), anti-SERCA_2_ antibody (Abcam, 1:1000 dilution), anti-RyR_2_ antibody (Abcam, 1:1000 dilution) or anti-Na^+^/Ca^2+^-exchangers (NCX_1_) antibody (Abcam, 1:1000 dilution). Appropriate secondary antibodies were used to detect the corresponding primary antibodies, and the antibody-antigen complexes in all membranes were detected by the ECL PLUS kit (Bio-Rad). The protein bands were quantified by Image Pro Plus 6.0.

### SERCA activity measurements

Samples of the left ventricle were prepared as the method previously described by Lizotte E [7]. The ATPase activity in samples was assessed with an ATPase activity assay kit (Nanjing Jiancheng Bioengineering Institute, Nanjing, China) based on a colorimetric estimation of ATP hydrolysis-produced inorganic phosphate. According to manufacturer’s instructions, the reactions were initiated by adding samples into the ATPase activity assay buffer. Following 10 min-reaction at 37°C, the contents of inorganic phosphate in samples were determined by optical densities (OD) measured with the Infinite M1000 PRO plate reader (Tecan, Switzerland) at 636 nm. The protein concentration in samples was determined by BCA kit (Pierce Company). The enzyme activity was defined as the contents of inorganic phosphorus (μmol) per mg total protein and hour.

### Statistical analysis

All statistical analyses were performed using the SPSS Statistical software (version 17.0). Values are expressed as mean ± standard error (SEM). One-way analysis of variance was used to analyze the differences between the means of groups, followed by Tukey post hoc test. A value of *P* <0.05 was considered significant.

## Results

### Establishment of hypertrophic model

Cardiac structure and function in rats were assessed by echocardiography at 4 weeks postsurgery (Additional file 1A). The parameters of LV wall thickness including IVSs, IVSd, LVPWs and LVPWd were significantly increased in aortic banded rats compared with sham rats (Additional file 1B). On the contrary, LVIDs was significantly decreased in aortic banded rats compared with sham rats, whereas no significant difference in LVIDd was detected between the two groups (Additional file 1C). Moreover, the parameters of systolic function (EF and FS) were significantly increased in banded rats compared to sham rats (Additional file 1D). These data indicated that the rat model of compensatory cardiac hypertrophy induced by aortic banding was successfully established.

### Antihypertrophic effect of resveratrol

**Figure 1:**
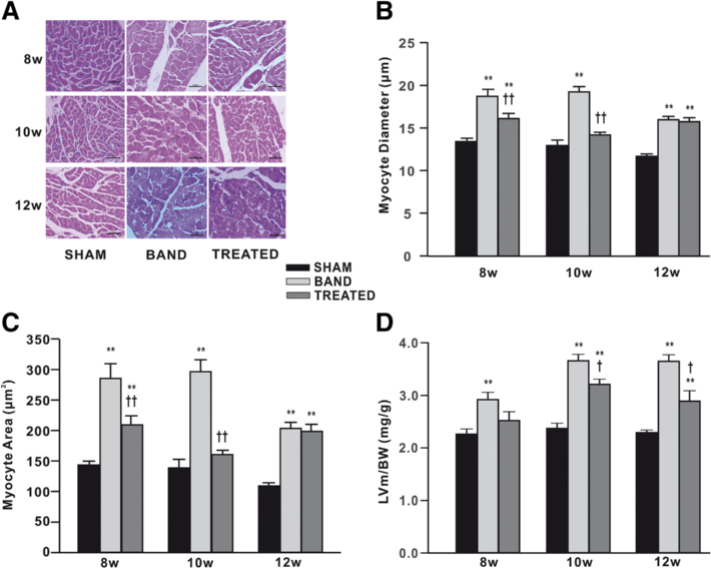
**Effects of resveratrol on the size of cardiomyocytes (n = 3) and the LV mass-to-body weight ratio (LVm/BW) (n = 6). (A)** Photomicrographs of left ventricular tissue sections stained by hematoxylin and eosin. **(B)** Myocyte diameter. **(C)** Myocyte area. **(D)** LVm/BW. Data are mean ± SEM. ^*^ *P* < 0.05 ^**^ *P* < 0.01 vs. sham rats; ^†^ *P* < 0.05 ^††^ *P* < 0.01 vs. banded rats.

Histological analysis showed that surface areas and diameters of cardiomyocytes were remarkably increased at 8, 10 and 12 weeks postsurgery in aortic banded rats compared with sham rats, but decreased by 27%, 14% at 8 weeks and 46%, 26% at 10 weeks postsurgery respectively in resveratrol-treated rats compared to banded rats. However, we found that resveratrol treatment failed to reverse enlargement in cardiomyocytes at 12 weeks postsurgery (Figure 1A-C).

The LVm/BW was increased by 29%, 54% and 59% in banded rats at 8, 10 and 12 weeks postsurgery respectively compared to sham rats. The LVm/BW in resveratrol-treated rats was not significantly different at 8 weeks postsurgery and significantly increased by 35% and 26% at 10 and 12 weeks postsurgery compared with sham rats, whereas was lower than banded rats at 10 and 12 weeks (Figure 1D).

### Effect of resveratrol on LV chamber

**Figure 2:**
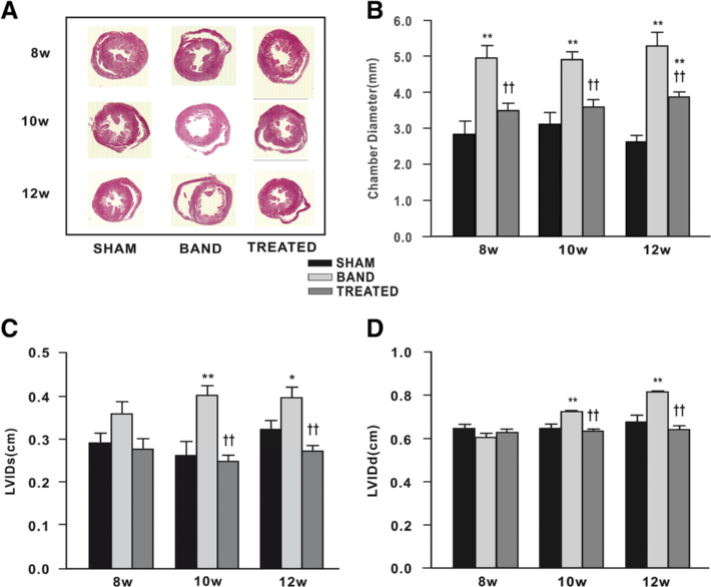
**Effect of resveratrol on left ventricular (LV) chamber (n = 4 ~ 7). (A)** Photomicrographs of the cross sectional area of LV chamber stained by hematoxylin and eosin. **(B)** Chamber diameter of LV. **(C)** LV internal dimensions at systole (LVIDs). **(D)** LV internal dimensions at diastole (LVIDd). Both LVIDs and LVIDd were detected by echocardiographic analysis. Data are mean ± SEM. ^*^ *P* < 0.05 ^**^ *P* < 0.01 vs. sham rats; ^†^ *P* < 0.05 ^††^ *P* < 0.01 vs. banded rats.

H&E staining analysis of cardiac cross-sections revealed dilation of LV in banded rats at 8, 10 and 12 weeks postsurgery. Resveratrol treatment showed a significant alleviation of LV dilation induced by aortic band at indicated time point (Figure 2A, B). Consistent with the histological data, LVIDd and LVIDs detected by echocardiographic analysis were also increased in banded rats compared with sham rats at 10 and 12 weeks postsurgery, and this effect was eliminated by resveratrol treatment (Figure 2C, D).

### Effect of resveratrol on cardiac systolic function

**Figure 3:**
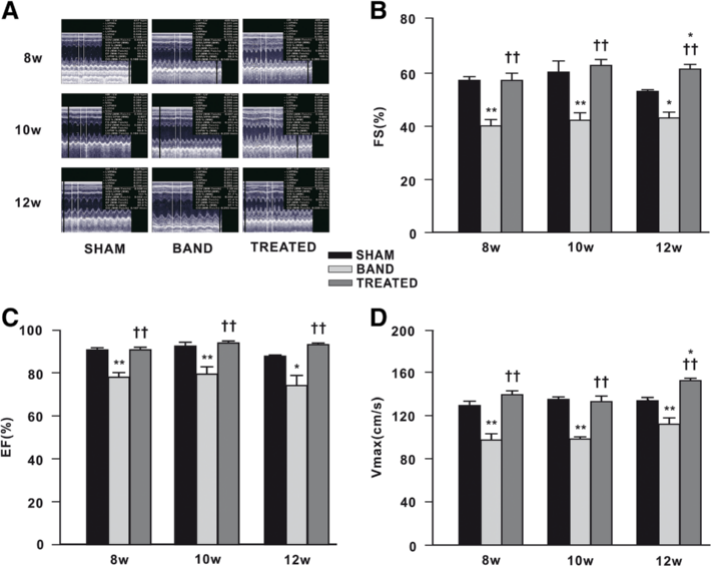
**Effect of resveratrol on left ventricular (LV) systolic function (n = 4 ~ 7). (A)** Representative pictures of echocardiography. **(B)** Fractional shortening (FS). **(C)** Ejection fraction (EF). **(D)** Maximal velocity through left ventricular outflow tract (Vmax). Data are mean ± SEM. ^*^ *P* < 0.05 ^**^ *P* < 0.01 vs. sham rats; ^†^ *P* < 0.05 ^††^ *P* < 0.01 vs. banded rats.

Echocardiographic analysis of cardiac function was carried out at 8, 10, and 12 weeks postsurgery in sham, banded and resveratrol-treated rats. We found that systolic functional parameters (EF, FS and Vmax) in banded rats were decreased continuously compared to sham rats at 8, 10, and 12 weeks postsurgery. Of note, resveratrol treatment significantly increased EF by 14%, 15%, 26%, FS by 30%, 32%, 26%, and Vmax by 30%, 26%, 25% at 8, 10, 12 weeks postsurgery respectively, compared with banded rats. FS and Vmax in resveratrol-treated rats were even remarkably higher than sham rats at 12 weeks postsurgery (Figure 3).

### Effect of resveratrol on cardiac fibrosis

**Figure 4:**
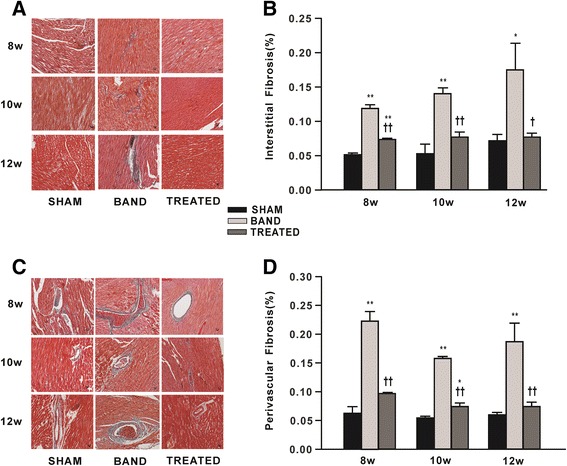
**Effect of resveratrol on cardiac fibrosis in left ventricular (LV) myocardium (n = 4). (A)** Representative photomicrographs of Masson trichrome staining for interstitial fibrosis showing myocardium in red and fibrosis in blue. **(B)** Quantification of interstitial fibrosis. **(C)** Representative photomicrographs of Masson trichrome staining for perivascular fibrosis. **(D)** Quantification of perivascular fibrosis. Data are mean ± SEM. ^*^ *P* < 0.05, ^**^ *P* < 0.01 vs. sham rats; ^†^ *P* < 0.05, ^††^ *P* < 0.01 vs. banded rats.

Fibrosis in the cross-sections of heart was stained blue with the Masson’s trichrome kit. We detected that both interstitial and perivascular fibrosis were increased in aortic banded rats at 8, 10 and 12 weeks postsurgery respectively, compared to sham rats. Interestingly, resveratrol treatment significantly reduced interstitial fibrosis by 38%, 45%, 56% and perivascular fibrosis by 56%, 53%, 60% at 8, 10 and 12 weeks postsurgery respectively, compared with banded rats, suggesting that resveratrol treatment can inhibit myocardial fibrosis caused by aortic banding (Figure 4).

### Effect of resveratrol on myocardium ultrastructure

**Figure 5:**
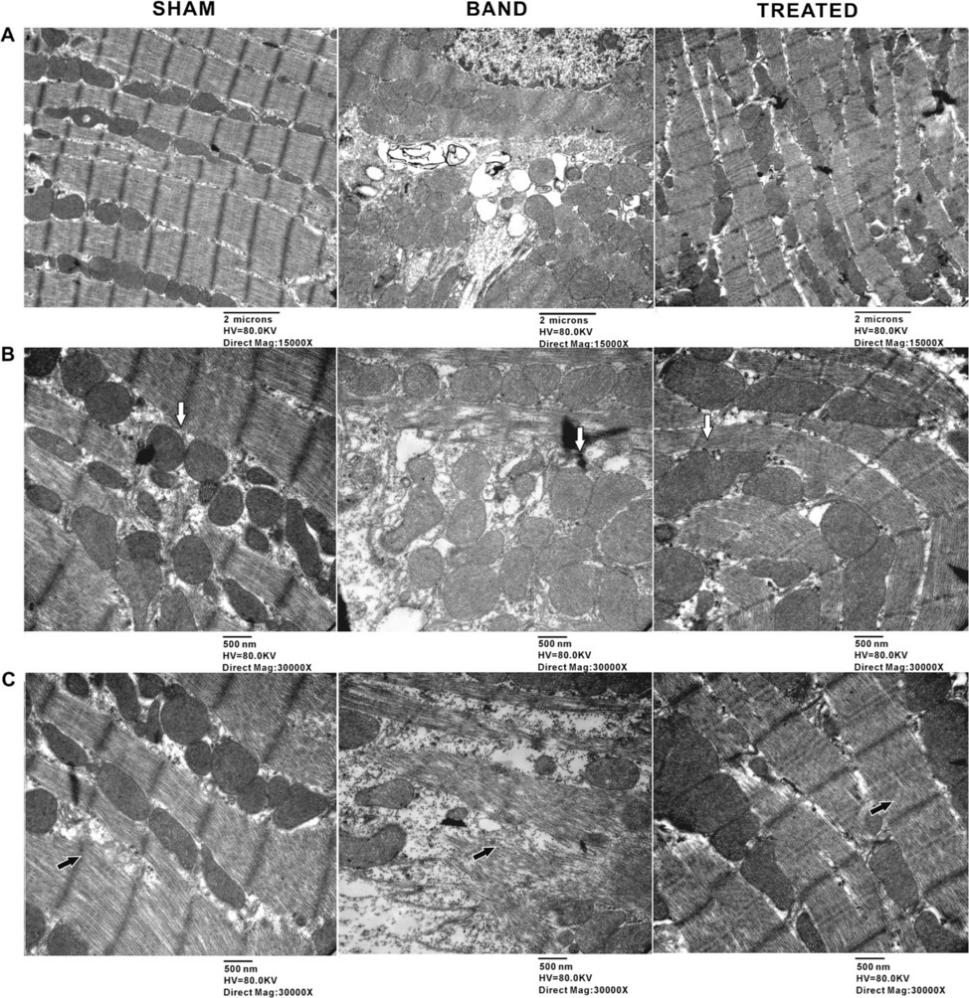
**Effects of resveratrol on myocardium ultrastructure detected by transmission electron microscopy (TEM).** Representative TEM image illustrating **(A)** ultrastructure of myocardium with 15000 × Magnification, **(B)** mitochondria (white arrow) and **(C)** myofilaments (black arrow) with 30000 × Magnification in sham, banded and resveratrol-treated rats.

TEM revealed the disrupted myocardium and disorganized mitochondria with abnormal cristae structure in banded rats at 12 weeks postsurgery (Figure 5). Cardiac muscle fibers from sham rats exhibited normal ultrastructural morphology, characterized by laterally aligned myofibrils with highly organized sarcomeres and elongated mitochondria packed tightly in strands running between myofibrils. However, in myocardium from banded rats, myofibrils were fragmented, disrupted and degraded, with disorganized arrays of sarcomeres. Mitochondria appeared rounded or irregular, extensive swelling, abnormally clumped or dispersed, and occasionally broken cristae. Some mitochondria were markedly degraded in cristae with lucent matrix and vacuolation. In myocardium from resveratrol-treated rats, the ultrastructure of cardiomyocytes was effectively maintained and nearly similar to those in sham rats, exhibiting slightly disrupted myofibrils. The mitochondria from resveratrol-treated rats appeared to be irregular shapes, obvious swollen and mildly clumped, but degraded cristae and vacuolations as shown in banded rats were not found in resveratrol-treated rats. Additionally, the apparent loss of myofibrils was only detected in hearts from banded, but not from resveratrol-treated rats (Figure 5).

### Effect of resveratrol on Ca^2+^ handling proteins

**Figure 6:**
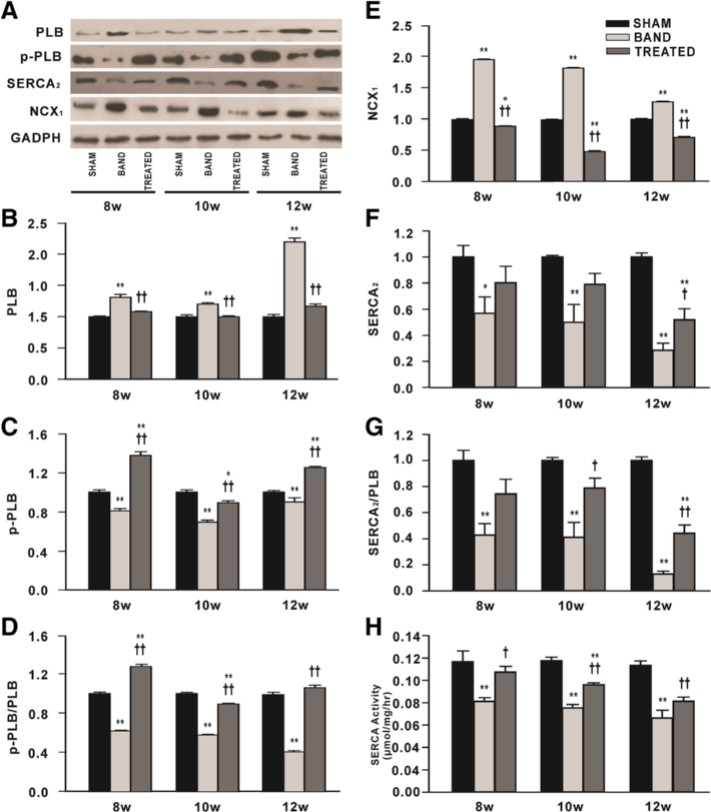
**Effects of resveratrol on the expressions of proteins responsible for SR Ca** ^**2+**^ **load and sarcoplasmic reticulum Ca** ^**2+**^ **-ATPase activity (n = 3). (A)** Representative western blot from each group. **(B)** The levels of Phospholamban (PLB). **(C)** The levels of Phospho-PLB (S16) (p-PLB). **(D)** The ratio of p-PLB to PLB. **(E)** The levels of Na^+^/Ca^2+^-exchangers (NCX _1_). **(F)** The levels of Sarcoplasmic reticulum Ca^2+^-ATPase (SERCA _2_). **(G)** The ratio of SERCA _2_ to PLB. **(H)** SERCA activity. Levels of proteins were quantified by densitometry and normalized against GADPH. Data are mean ± SEM. ^*^ *P* < 0.05 ^**^ *P* < 0.01 vs. sham rats; ^†^ *P* < 0.05 ^††^ *P* < 0.01 vs. banded rats.

The levels of total PLB in hearts from banded rats were markedly increased compared with sham rats, and enhanced by 2.2-fold especially at 12 weeks postsurgery. Resveratrol treatment continuously alleviated the increased total PLB expression. On the contrary, the levels of p-PLB which were normalized by the levels of GADPH or total PLB were remarkably decreased at 8, 10 and 12 weeks postsurgery in banded rats relative to sham rats, this effect was prevented by resveratrol treatment (Figure 6A-D).

The levels of NCX_1_ were increased continuously in banded rats compared with sham rats. Resveratrol completely prevented the upregulation of NCX_1_ induced by aortic band and even significantly lowered it relative to sham group (Figure 6A, E). On the contrary, SERCA_2_ levels were markedly decreased in banded rats compared with sham rats at 8, 10 and 12 weeks postsurgery, accompanied with decreased ratio of SERCA_2_ to PLB and SERCA activity. Notably, resveratrol treatment enhanced the levels of SERCA_2_ and the ratio of SERCA_2_ to PLB, and prevented the decreased SERCA activity caused by aortic banding (Figure 6A, F-H).

**Figure 7:**
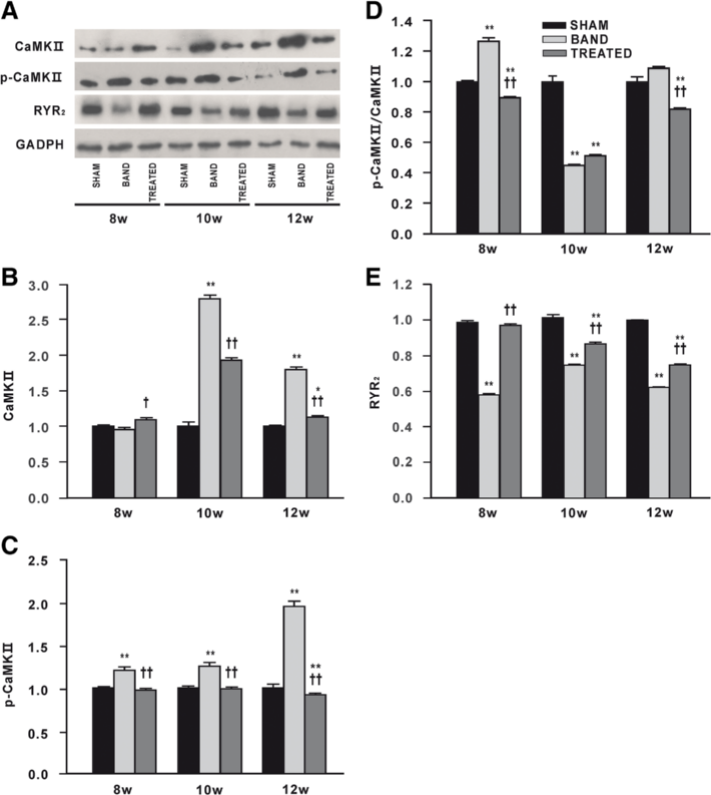
**Effects of resveratrol on protein expressions of Ca** ^**2+**^ **/calmodulin-dependent protein kinase II (CaMK II), phospho-CaMKII (Thr286) (p-CaMK II) and ryanodine receptor (RyR**_**2**_ **) (n = 3). (A)** Representative western blot from each group. **(B)** The levels of CaMK II. **(C)** The levels of p-CaMK II. **(D)** The ratio of p-CaMK II to CaMK II. **(E)** The levels of RyR_2_. Levels of proteins were quantified by densitometry and normalized against GADPH. Data are mean ± SEM. ^*^ *P* < 0.05 ^**^ *P* < 0.01 vs. sham rats; ^†^ *P* < 0.05 ^††^ *P* < 0.01 vs. banded rats.

The levels of total CaMKII in hearts from banded rats were unchanged at 8 weeks postsurgery, but increased significantly at 10 and 12 weeks postsurgery compared with sham rats. Resveratrol remarkably alleviated the elevation of total CaMKII induced by aortic banding. Accordingly, the levels of p-CaMKII were progressively increased at 8, 10 and 12 weeks postsurgery in banded rats, which were completely prevented by resveratrol (Figure 7A-C). However, due to sharply rising of total CaMKII, the ratio of p-CaMKII to total CaMKII in banded rats was no different with sham rats at 12 weeks postsurgery, and even lower at 10 weeks postsurgery (Figure 7A, D). Meanwhile, the significant decreases in RyR_2_ levels were observed in banded rats compared with sham rats at 8, 10 and 12 weeks postsurgery, and the reduction of RyR_2_ levels induced by aortic banding was prevented by resveratrol treatment (Figure 7A, E).

## Discussion

This study provides several new findings showing that resveratrol prevents the transition from cardiac hypertrophy to HF induced by PO. First, we demonstrated resveratrol reversed LV hypertrophy induced by PO with time limitation. Second, we showed that resveratrol strongly and continuously prevented the cardiac structural and functional exacerbation due to PO. Third, we showed that resveratrol exerted multiple actions that may contribute to the beneficial effects including (1) inhibition of cardiac fibrosis, (2) prevention of myocardium ultrastructural alterations, (3) modulation of activation and expression of Ca^2+^ handling proteins.

### Antihypertrophic effect of resveratrol has a time limit

Antihypertrophic effect of resveratrol and its analogue has been documented in different experimental settings of PO [1,3,4,8-12]. In this study, 4 weeks after aortic banding surgery, rats developed PO-induced centric hypertrophy, as indicated by significant increase in wall thickness without chamber dilation. Consistent with previous studies, resveratrol treatment regressed developed cardiac hypertrophy at 8 and 10 weeks postsurgery. However, resveratrol did not prevent cardiac hypertrophy at the late stage of the experiment, as shown by histological analysis. The data of LVm/BW also suggested that the effect of resveratrol was receded gradually as PO was sustained. Developed cardiac hypertrophy in treated rats was further confirmed by echocardiography analysis, which showed a marked increase in wall thickness compared with sham rats at 12 weeks postsurgery (see Additional file 2). This novel finding indicated that the timing of treatment is a critical factor for antihypertrophic effect of resveratrol.

### Resveratrol prevented the development of decompensatory phase

4-week aortic banding caused compensatory centric hypertrophy characterized by high contractile function of LV, increased thickness of LV wall and decreased LVIDs. However, with long-standing untreated PO, LV systolic performance decreased and LV dilatation occurred, which are the key features of decompensatory phase, namely HF. Resveratrol treatment effectively preserved cardiac systolic function and LV chamber dimension. Previous studies demonstrated that resveratrol improved contractile function with abolishing cardiac hypertrophy caused by PO [1,3]. Interestingly, we found that resveratrol continuously exerted cardioprotective effects in banded rats and these effects sustained to decompensatory stage of HF even though its antihypertrophic effect was absent. Similarly, Ste’phanie Rimbaud found that resveratrol treatment exerted beneficial protective effects on survival and cardiac contraction in DSS rats fed with HS-NT which is a hypertensive model of HF [5]. However, the mechanism underlying the development of HF in DSS rats is apparently different from aortic banded rats.

### Cardioprotective mechanisms of resveratrol

The development of HF is associated with marked myocardial fibrosis which is characterized by excessive extracellular matrix (ECM) deposition and myocardial stiffness [13]. Limiting pathological myocardial fibrosis represents a potential therapeutic target to prevent HF. Previous studies have demonstrated that resveratrol has beneficial effects to reduce cardiac fibrosis in a variety of pathological models [14,15]. For instance, resveratrol alleviated cardiac fibrosis in spontaneously hypertensive rats [16] and decreased left ventricular interstitial and perivascular fibrosis in DOCA-salt rats [17]. We demonstrated that resveratrol inhibited interstitial and perivascular fibrosis due to PO, indicative of the improvement of myocardial compliance and diastolic function. Indeed, increased LV pressure half-time, an index of diastolic heart function, was detected in banded rats, but prevented by resveratrol (see Additional file 3). Meanwhile, inhibition of cardiac fibrosis by resveratrol could explain the findings that LVm/BW in treated rats was remarkably lower than in banded rats at 12 weeks postsurgery while no difference in the size of cardiomyocytes was detected by histological analysis between two groups.

Second, we found that resveratrol protected against myocardial ultrastructural abnormalities induced by PO. The most obvious detriment to myocardial ultrastructure induced by PO was decrease of myofibrils as a result of rupture and degradation of myofilaments. Notably, resveratrol treatment normalized myocardial ultrastructure as evidenced by intact myofibrils and organized sarcomeres, which may be attributed to multiple mechanisms beneficial to cell survival including prevention of cardiomyocyte apoptosis, regulation of autophagy, and reduction of oxidative stress [14,18-20]. The other striking abnormality caused by PO was swollen and disordered mitochondria revealed by TEM. Resveratrol only partially prevented mitochondrial lesions. However, other studies demonstrated that resveratrol significantly attenuated the abnormality of mitochondrial ultrastructure in sepsis-induced myocardial depression with a large dose (30 mg/kg or 60 mg/kg) [21], and preserved mitochondrial function and biogenesis contributing to improve cardiac energy metabolism and reduce oxidative stress in other models [5,20,22,23]. This difference could be explained by the diversity of resveratrol dosage, the period of treatment, the stage of HF and animal models.

Ca^2+^ cycling which refers to the release and reuptake of intracellular Ca^2+^ is highly regulated in cardiomyocytes and determines the process of cardiac muscle contraction and relaxation. Defects in the regulation of Ca^2+^ handling proteins contribute to HF. To our knowledge, the effects of resveratrol on Ca^2+^ handling proteins in HF remain unclear. Impaired SERCA_2_ function and enhanced NCX activity have been proposed as causes of reduced SR Ca^2+^ load in HF. Moreover, PLB, a regulator of the affinity of SERCA_2_ for Ca^2+^, balances SR Ca^2+^ uptake through inhibiting the affinity of SERCA_2_ for Ca^2+^ by unphosphorylated PLB and relieving this inhibition by phosphorylation. As a negative regulator of SERCA_2_, hypophosphorylated PLB is an important cause of deficient SR Ca^2+^ uptake in failing hearts. Accordingly, both SERCA_2_ overexpression and PLB inhibition have been designed as therapeutic strategies for HF [6]. In this study, PO caused substantially decreased SERCA_2_ expression and ATPase activity, elevated NCX_1_, increased PLB and decreased p-PLB, indicating that SR Ca^2+^ load in this animal model is deficient, consequently leading to impaired contractile function. Resveratrol effectively upregulated SERCA_2_ levels, ATPase activity and the ratio of phosphorylated to unphosphorylated PLB, accompanied by a significant downregulation of NCX_1_, indicative of improved SR Ca^2+^ load in hearts. Consistent with our results, a previous study showed that increased SERCA_2a_ expression by resveratrol improved contractile function in chronic type 1 diabetes [12].

Additionally, we detected a significant increase in expression and phosphorylation of CaMKII due to PO. It has been reported that CaMKII hyperphosphorylation of RyR_2_ accounts for excessive diastolic SR Ca^2+^ leak in non-ischemic (aortic banding) cardiomyopathy, but not in ischemia (post-MI) in which PKA phosphorylation of RyR_2_ is involved [24]. This leads to increased RyR_2_ open probability and a diastolic SR Ca^2+^ leak because of a higher sensitivity to Ca^2+^-induced Ca^2+^ release at low cytoplasmic Ca^2+^ concentrations. In our experiment, resveratrol attenuated the increase in CaMKII level and completely inhibited hyperphosphorylation of CaMKII. It has been demonstrated that RyR_2_ leak was inhibited through inhibition of CaMKII phosphorylation. Knock-in mice with an inactivated CaMKII phosphorylation site on RyR_2_ had lower SR Ca^2+^ leak and improved SR Ca^2+^ load, and were relatively protected from HF development after transverse aortic constriction [25]. In patients with HF, CaMKII- but not PKA-dependent RyR_2_ phosphorylation was significantly increased, accompanied by increased SR Ca^2+^ leak, reduced systolic Ca^2+^ transients, depletion of SR Ca^2+^ storage and elevated diastolic Ca^2+^ levels. Moreover, CaMKII inhibition, but not inhibition of PKA yielded a reduction of the SR Ca^2+^ leak [25]. Therefore, we speculate that CaMKII downregulation by resveratrol plays a major role in preventing the development of cardiac dysfunction in aortic banded animals via inhibition of RyR_2_ leak.

Although the activity of RyR_2_ channel in HF was investigated in many studies, very little is known about expression of RyR_2_ and its significance in failing heart. We found that RyR_2_ protein expression was significantly decreased in myocardium of HF and resveratrol effectively enhanced the expression of RyR_2_. We speculate that downregulation of RyR_2_ may decrease systolic Ca^2+^ release and impair cardiac contractility. Similarly, Kubalova et al. found that RyR_2_ content in failing hearts was decreased to approximately half of control values, but the levels of other proteins of the Ca^2+^ release channel complex such as triadin and junctin were not changed. The altered stoichiometry of triadin and junctin to RyR_2_ may increase activity of RyR_2_, thus leading to the abnormal Ca^2+^ handling in HF [26].

A prevailing theory is that one of the major mechanisms proposed to underlie resveratrol mediated cardioprotection is reduction of oxidative stress [4,27,28]. Polydatin, a resveratrol glucoside, has been shown to prevent enhanced Ca^2+^ spark-mediated SR leak by reducing oxidative stress in RyR_2_ in burn-traumatized heart, leading to protection of cardiac function against burn injury [29]. Further studies are needed to understand whether reduction of oxidative stress by resveratrol could regulate RyR_2_ and other Ca^2+^ handling proteins in aortic banded rats.

## Conclusions

These data demonstrated that resveratrol treatment prevented cardiac hypertrophy induced by PO with time-limitation and inhibited the development of HF via improving cardiac structure and function. The novel findings on mechanisms underlying cardioprotection of resveratrol include protection of myocardium ultrastructure and regulation of global Ca^2+^ handling proteins.

## Additional files

Additional file 1:
**Two-dimensionally guided M-mode echocardiographic measurement of wall thickness (IVSs, IVSd, LVPWs and LVPWd) (A and B), chamber dimensions (LVIDs and LVIDd) (A and C), systolic parameters (FS and EF) (A and D) of left ventricular (LV) in sham and banded rats at 4 weeks postsurgery (n = 6~13).** Data are mean±SEM. ^*^ *P*<0.05 ^**^ *P*<0.01 vs. sham rats. Additional file 2:
**Two-dimensionally guided M-mode echocardiographic measurement of IVSd (A), IVSs (B), LVPWd (C) and LVPWs (D) in sham, banded and resveratrol-treated rats at 8, 10 and 12 weeks postsurgery (n = 4~7).** Data are mean±SEM. ^*^ *P*<0.05 vs. sham rats; ^†^ *P*<0.05 vs. banded rats. Additional file 3:
**Effect of resveratrol on left ventricular (LV) cardiac output and pressure half-time (n = 4~7).** (A) Representative pictures of echocardiography. (B) Cardiac output. (C) Pressure half-time. Data are mean±SEM. ^*^ *P*<0.05 ^**^ *P*<0.01 vs. sham rats; ^†^ *P*<0.05 ^††^ *P*<0.01 vs. banded rats.

## Abbreviations

HF: Heart failure
PO: Pressure overload
SR: Sarcoplasmic reticulum
LV: Left ventricular
FS: Fractional shortening
EF: Ejection fraction
Vmax: Maximal velocity through left ventricular outflow tract
LVIDd and LVIDs: LV internal dimensions at both diastole and systole
LVPWd and LVPWs: LV posterior wall dimensions at both diastole and systole
IVSd and IVSs: Interventricular septal dimensions at both diastole and systole
LVm/BW: LV mass-to-body weight ratio
TEM: Transmission electron microscopy
CaMKII: Ca^2+^/calmodulin -dependent protein kinase II
p-CaMKII: Phospho-CaMKII (Thr286)
SERCA_2_: Sarcoplasmic reticulum Ca^2+^ ATPase _2_
RyR_2_: Ryanodine receptor _2_
PLB: Phospholamban
NCX_1_: Na^+^/Ca^2+^-exchangers
p-PLB: Phospho-PLB (S16)
