# Supplementary figures and images for: Resveratrol ameliorates cardiac dysfunction induced by pressure overload in rats via structural protection and modulation of Ca2+ cycling proteins
Source: J Transl Med. 2014 Nov 26;12:323. doi: 10.1186/s12967-014-0323-x (PMC4278670; doi:10.1186/s12967-014-0323-x)

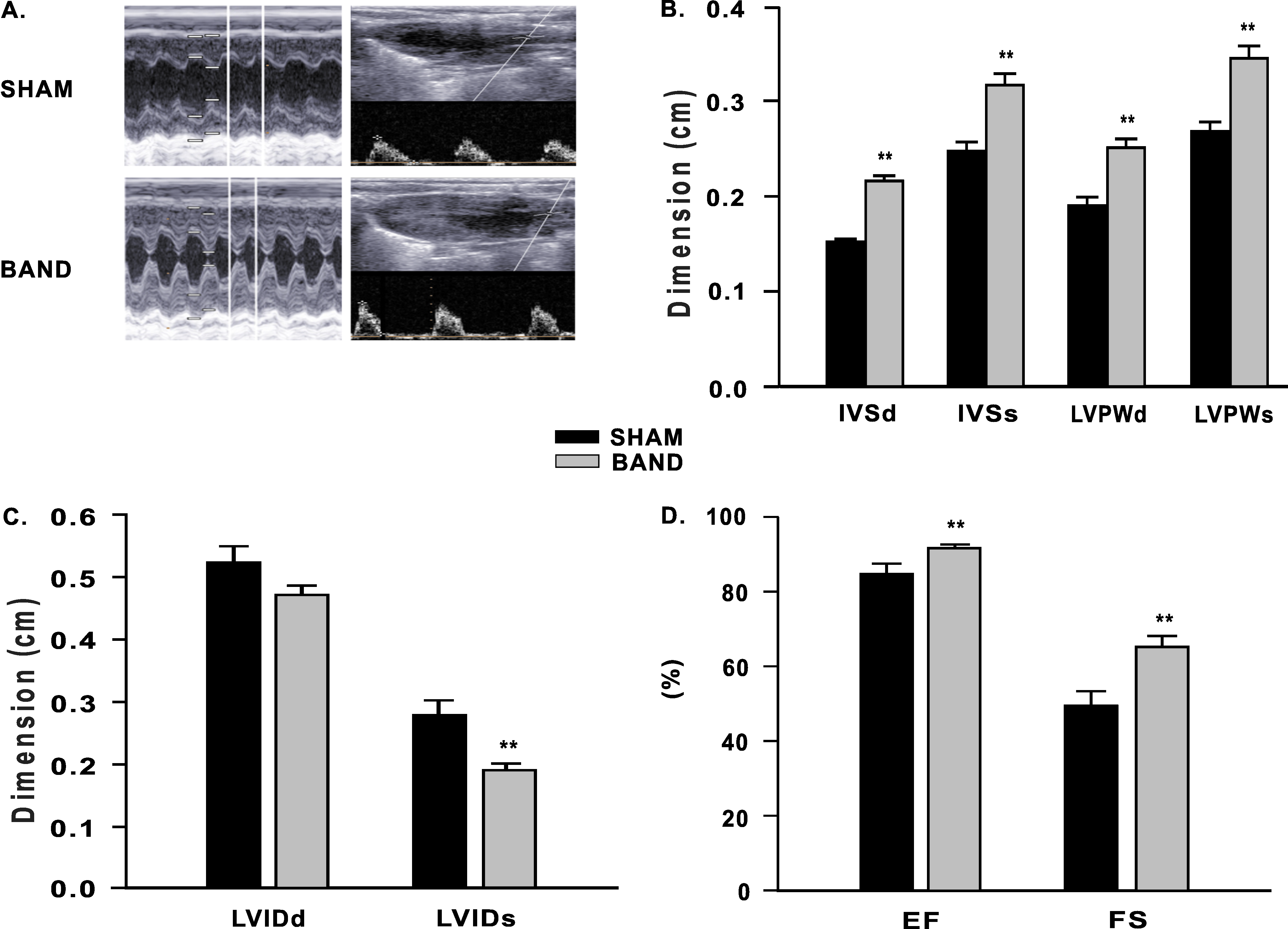

Supplement: Additional file 1: — Two-dimensionally guided M-mode echocardiographic measurement of wall thickness (IVSs, IVSd, LVPWs and LVPWd) (A and B), chamber dimensions (LVIDs and LVIDd) (A and C), systolic parameters (FS and EF) (A and D) of left ventricular (LV) in sham and banded rats at 4 weeks postsurgery (n = 6~13). Data are mean±SEM. * P<0.05 ** P<0.01 vs. sham rats. [file 12967_2014_323_MOESM1_ESM.tiff]

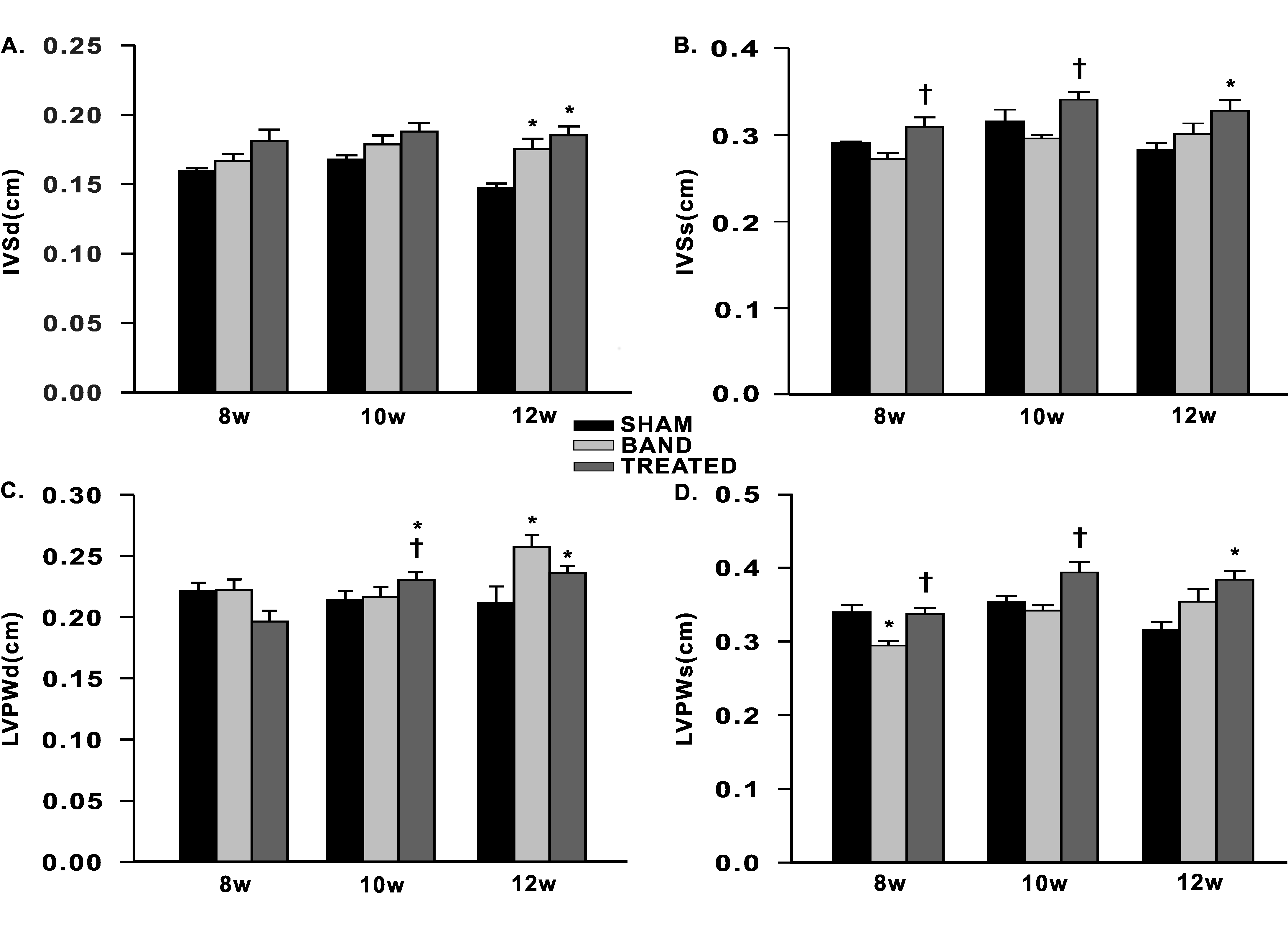

Supplement: Additional file 2: — Two-dimensionally guided M-mode echocardiographic measurement of IVSd (A), IVSs (B), LVPWd (C) and LVPWs (D) in sham, banded and resveratrol-treated rats at 8, 10 and 12 weeks postsurgery (n = 4~7). Data are mean±SEM. * P<0.05 vs. sham rats; † P<0.05 vs. banded rats. [file 12967_2014_323_MOESM2_ESM.tiff]

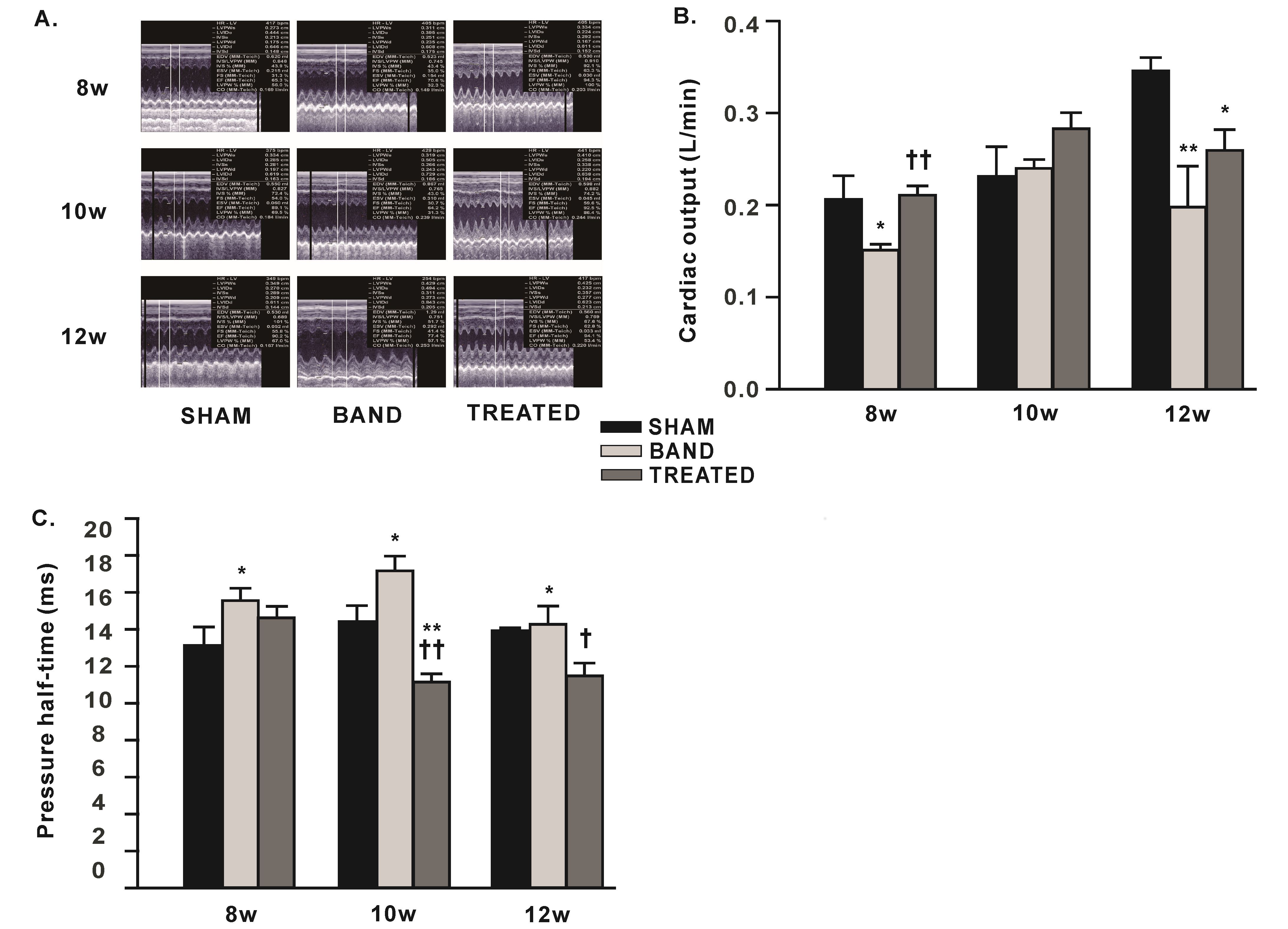

Supplement: Additional file 3: — Effect of resveratrol on left ventricular (LV) cardiac output and pressure half-time (n = 4~7). (A) Representative pictures of echocardiography. (B) Cardiac output. (C) Pressure half-time. Data are mean±SEM. * P<0.05 ** P<0.01 vs. sham rats; † P<0.05 †† P<0.01 vs. banded rats. [file 12967_2014_323_MOESM3_ESM.tiff]
